# Effect of environmental salinity on expression of adrenomedullin genes suggests osmoregulatory activity in the medaka, *Oryzias latipes*

**DOI:** 10.1186/s40851-015-0012-5

**Published:** 2015-03-10

**Authors:** Maho Ogoshi, Kanoko Kato, Tatsuya Sakamoto

**Affiliations:** Ushimado Marine Institute, Faculty of Science, Okayama University, 130-17 Kashino, Ushimado, Okayama 701-4303 Japan

**Keywords:** Adrenomedullin, Osmoregulation, Medaka

## Abstract

**Introduction:**

The adrenomedullins (AMs) comprise a hormonal family in mammals and teleost fishes, with five members (AM1–5) found or predicted in most of the teleosts including Japanese medaka (*Oryzias latipes*). AM1 is known to have cardiovascular and osmoregulatory functions in mammals, but the roles of most AMs are yet to be determined.

**Results:**

Using medaka, we first analyzed the tissue distribution of all five AM genes and found detectable expression in all tissues examined, with relatively high levels of AM3 and AM5 in the liver and kidney. To assess the osmoregulatory roles of these AMs, mRNA levels were examined in the brain (including the eyes), gill, liver, kidney and spleen of medaka one week after transfer from isotonic saline (11 ppt) to freshwater (0 ppt) or seawater (33 ppt). Expression of AM1 in the brain-eye increased in freshwater. The central level of AM4 (the paralog of AM1) decreased in seawater; the branchial level of AM4 decreased in freshwater and seawater, but the renal level increased in freshwater. The branchial level of AM2 increased in seawater, whereas the renal level decreased in freshwater and seawater. Expression of AM3, the AM2 paralog, decreased in the brain-eye of seawater-acclimated fish. Expression of AM5 in the brain-eye and kidney decreased in seawater.

**Conclusions:**

Except for branchial AM2, the members of AM family tend to be involved in promotion of hyper-osmoregulation and/or inhibition of hypo-osmoregulation, although each AM may play a distinct role during adaptation to different salinities.

## Introduction

Adrenomedullin (AM) is a peptide hormone that was first isolated from human pheochromocytoma in 1993 [1]. In mammalian species, AM (AM1) consists of 52 amino acids with a ring structure formed by a single disulfide bond between two cysteine residues. AM1 was initially thought to be a member of the calcitonin gene-related peptide (CGRP) family, together with CGRP and amylin. However, our group discovered five distinct AMs (AM1–5) in the pufferfish, *Takifugu rubripes* [2] and, as orthologs, AM2 and AM5 in mammals [3,4]. AM2 was also identified at the same time by another group, and named intermedin [5]. Five AMs have also been identified in medaka (*Oryzias latipes*) and four in Japanese eel (*Anguilla japonica*) [3,6]. Comparative genomic analyses revealed that these AMs evolved from three ancestral genes, and thus can be divided into three groups: AM1/AM4, AM2/AM3 and AM5 [3]. Based on these results, AMs are considered to comprise a subfamily within the CGRP family.

AM1 has multiple biological effects in mammals, including regulation of body fluid (hypotension, diuresis and natriuresis), bronchodilation, regulation of secretion of other hormones, and antimicrobial effects [1,7-11]. AM2 and AM5 also induce hypotension in rodents [4,12], but the other functions of these peptides are unknown, despite their widespread distribution in mammalian tissues. In non-mammalian species, AMs have only been characterized in teleost fishes, in which they are hypothesized to be involved in body fluid regulation and osmoregulation, as in mammals, since administration of AM2 or AM5 induces potent hypotension and water intake in eel [6,13]. However, the role of these endogenous peptides in osmoregulation is still unclear, especially in other teleost species.

All five genes of the AM family have been identified only in pufferfish and medaka. The medaka is a euryhaline teleost fish whose genome has been mapped and is used as a good model not only in developmental research [14-16] but also in studies of osmoregulation [17-20]. In this study, we first investigated the tissue distribution of mRNAs for the five AM genes in medaka. Then, to assess the role of AMs in osmoregulation, we analyzed the changes in mRNA levels of these genes after acclimation of medaka from isotonic 11 ppt saline to freshwater or 33-ppt seawater for one week, since previous studies by our group indicated that this species is suitable for use as a model in studies of osmoregulation, and many osmoregulatory/hormonal changes occurred at this time point in this species with the limited (statistically insignificant) size and growth differences, such as the adjustment of muscle water content (plasma osmolality, sodium and chloride) and the activation of branchial chloride cells in seawater, as well as the reduced Na^+^/K^+^-ATPase activities and the increased rate of routine oxygen consumption in freshwater, together with the regulation of cortisol-glucocorticoid receptor axis which is one of the central endocrine systems to the teleost osmoregulation [20-23].

## Materials and methods

### Animals

Adult fish of the orange-red variety of Japanese medaka (0.2–0.3 g) of both sexes were purchased from a local dealer (Fish Box 344, Okayama, Japan) and kept in an indoor freshwater tank at 25°C under a 12-h light/12-h dark photoperiod. Fish were fed 3% of their total weight once daily with Tetrafin flakes (Tetra Werke, Melle, Germany). To analyze the tissue distribution of AM mRNAs, the brain (including the eyes), gills, intestine, liver, kidney, and spleen were removed from at least two fish and snap-frozen in liquid nitrogen. Before handling, fish were anaesthetized with 0.01% tricaine methane sulfonate (Sigma, Tokyo, Japan) neutralized with sodium bicarbonate. All procedures were conducted with the approval of the Animal Care and Use Committee of Okayama University and in accordance with the Guidelines for Animal Experimentation established by the committee.

### Transfer experiment

Male fish of about the same size were acclimated to isotonic 11 ppt saline (149 mM Na, 176 mM Cl, 3.8 mM Ca, 346 mOsml/kg) for at least two weeks before use. They were divided into three groups (20 fish per group) and transferred to a plastic aquaria filled with 10 L of water at salinities of 0 ppt (freshwater), 11 ppt (control) or 33 ppt (seawater). Half of the water was changed in each tank every day. The brain-eyes (head except gills), gills, liver and kidney were collected one week after the transfer, the time point at which medaka growth is not influenced by salinity [21].

### Expression of AM genes

Total RNA was isolated from the frozen tissues using an RNeasy Plus mini-kit (Qiagen, Venlo, Netherlands) following the manufacturer’s instructions. Extracted total RNA (1 μg) was reverse-transcribed using an iScript cDNA Synthesis kit (Bio-Rad Laboratories, Hercules, CA, USA). The expression levels of AM1-5 were estimated by quantitative real-time PCR (MiniOpticon™ Real-Time PCR Detection System, Bio-Rad). The primers were designed from medaka AM genes and the ribosomal protein L7 (RPL-7) housekeeping gene [24] using Primer Express (Applied Biosystems, Foster City, CA, USA) and Primer3 software (Table 1). All medaka AM genes have been previously identified [3] (accession numbers: AM1, AB257074; AM2, AB257075; AM3, AB257076; AM4, AB257077; AM5, AB257078). The PCR reaction mixture contained 1 μl of cDNA (500 ng), 2 μl of primer mixture (both forward and reverse, 10 μM), 22 μl of DEPC-H_2_O and 25 μl of 2x iQ SYBR Green Supermix (Bio-Rad; 25 U/ml iTaq DNA polymerase, 20 mM Tris–HCl, pH 8.4, 50 mM KCl, 3 mM MgCl_2_, 200 μM dNTPs, SYBR Green I and 10 nM fluorescein). The following PCR cycle was used for gene amplification: one cycle at 95°C for 5 min; 40 cycles for denaturing at 95°C for 11 sec, annealing for 33 sec and extension at 65°C for 1 min; melting curve from 65°C to 95°C, followed by a 4°C hold at the end of the PCR. The annealing temperatures were 58°C for AM1, AM2 and AM5; 60°C for AM3 and AM4. The dissociation curves of the primer pairs showed a single peak. Samples were run in duplicate. Efficiency of the reaction was measured by the slope of a standard curve derived from serial dilutions of PCR products of each AM gene. The transcript abundances were normalized to RPL-7 mRNA expression, which was relatively constant (brain-eye: *P* = 0.26–0.88, gill: *P* = 0.22–0.74, liver: *P* = 0.22–0.72, kidney: *P* = 0.52–0.10 by Tukey-Kramer multiple comparison test following one-way analysis of variance [ANOVA]), as also shown previously [24].

**Table 1:** **Primers used for real**-**time PCR**

**Primer**	**Sequence**
Medaka AM1-F	AGGAGCAAAAATGGCAAGAA
Medaka AM1-R	ATCCCTGATGTCCTCTGGTG
Medaka AM2-F	CGAGTCTGACCTCTCGCTTT
Medaka AM2-R	ATGGCTGAGGTTCTGCACTT
Medaka AM3-F	TGTCTCCCAACACAGCAGAG
Medaka AM3-R	GGTTCTGGACTTGGCATGTT
Medaka AM4-F	AGGACAGCTCCATCCACATC
Medaka AM4-R	CCGTCCGTAACCATTTATGC
Medaka AM5-F	GTCTGAGGTGGGCGTTACAT
Medaka AM5-R	TTGGCGAGGTTGTGTAGTTG

### Statistical analysis

All analyses were performed with KyPlot software (KyensLab, Tokyo, Japan) using ANOVA and a post hoc Tukey-Kramer or Steel-Dwass test.

## Results

**Figure 1:**
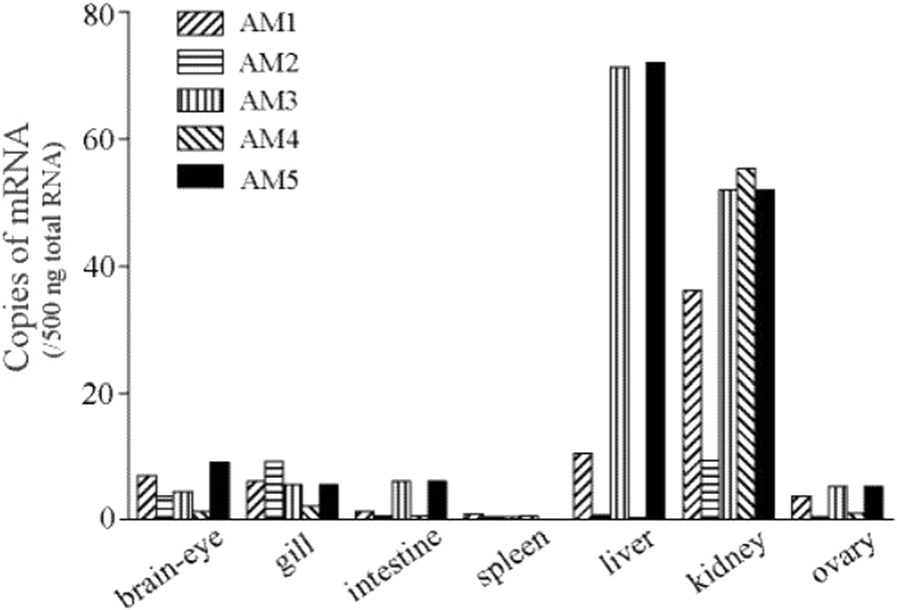
**Expression of adrenomedullin (AM) family genes in various tissues of freshwater medaka.** Values are means for at least two fish, except those for spleen, which were determined after pooling tissue from three fish, as expression levels were undetectable in individual fish. The expression level of AM5 in the spleen was 0.004 copies.

The tissue distribution of mRNAs for AM genes is shown in Figure 1. All AM genes were expressed at detectable levels in all tissues examined, but with notable expression in the liver and kidney. All genes except AM2 showed high expression in the kidney. In the liver, the mRNA levels for AM3 and AM5 were higher than those in other tissues.

**Figure 2:**
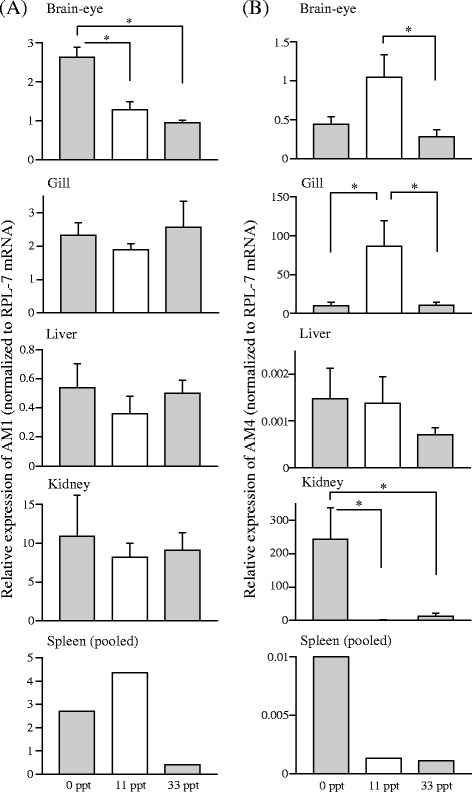
**Messenger RNA expression of AM1 (A) and AM4 (B) in tissues of medaka one week after transfer from an 11 ppt isotonic saline solution to salinities of 0 ppt (freshwater) and 33 ppt (seawater).** Asterisks indicate significant differences (*P < 0.05) in a Tukey-Kramer multiple comparison test following one-way ANOVA (**A**; **B**: brain and gill) or a Steel-Dwass test (**B**: kidney). Values are means ± SEM (*n* = 4–6) and mRNA levels are expressed in arbitrary units normalized to ribosomal protein L7 levels.

**Figure 3:**
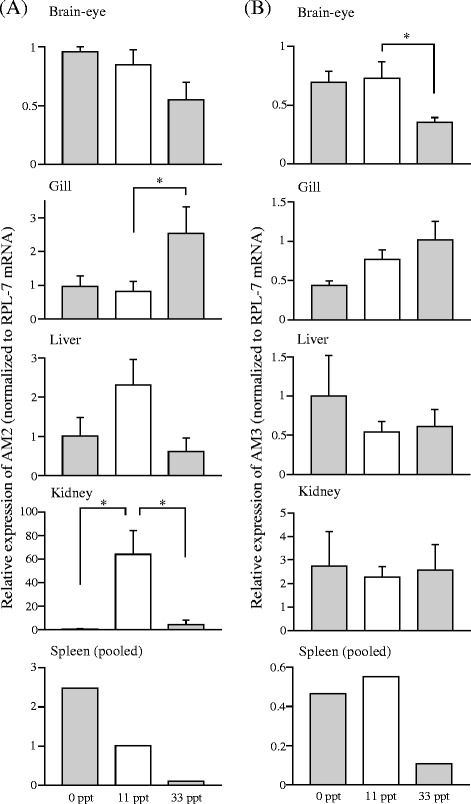
**Messenger RNA expression of AM2 (A) and AM3 (B) in tissues of medaka one week after transfer from an 11 ppt isotonic saline solution to salinities of 0 ppt (freshwater) and 33 ppt (seawater).** Asterisks indicate significant differences (*P < 0.05) in a Tukey-Kramer multiple comparison test following one-way ANOVA **(A)** or a Steel-Dwass multiple comparison test **(B)**. Values are means ± SEM (*n* = 4–6) and mRNA levels are expressed in arbitrary units normalized to ribosomal protein L7 levels.

**Figure 4:**
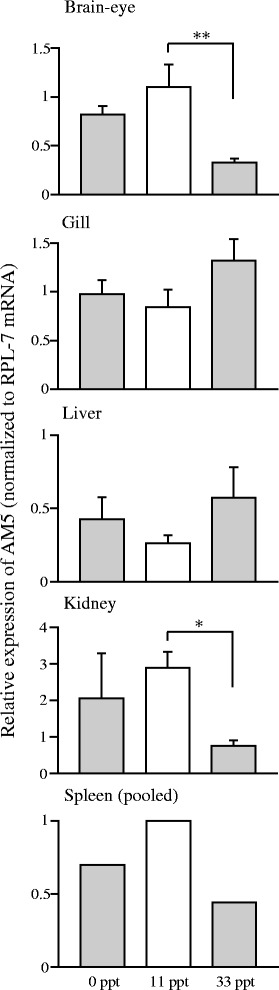
**AM5 mRNA expression in tissues of medaka one week after transfer from an 11 ppt isotonic saline solution to salinities of 0 ppt (fresh water) and 33 ppt (seawater).** Asterisks indicate significant differences in a Tukey-Kramer multiple comparison test following one-way ANOVA (**P < 0.01 in brain) or a Steel-Dwass’ test (*P < 0.05 in kidney). Values are means ± SEM (*n* = 4–6) and mRNA levels are expressed in arbitrary units normalized to ribosomal protein L7 levels.

The relative expression levels of AM genes in several tissues after the transfer from 11 ppt saline to 0 ppt freshwater or 33 ppt seawater are shown in Figures 2, 3 and 4. Expression of AM1 in brain-eye significantly increased in freshwater compared with 11 ppt control (Figure 2A). The mRNA level of AM4, the duplicated counterpart of AM1 [3], decreased significantly in the brain-eye after transfer to 33 ppt seawater, and those in the gill decreased as well in freshwater and seawater. In the kidney, the AM4 mRNA level was much higher in fish transferred to freshwater, compared to the levels in 11 ppt control and seawater (Figure 2B). The AM2 mRNA level in the gill was significantly higher in 33 ppt seawater than in 11 ppt control, whereas the AM2 mRNA level in the kidney was low in freshwater and seawater relative to that in 11 ppt control (Figure 3A). Expression of AM3, the duplicated paralog of AM2 [3], significantly decreased in the brain-eye in seawater compared with 11 ppt control (Figure 3B). AM5 expression in the brain-eye and kidney of fish in 33 ppt seawater was significantly lower than that in 11 ppt control (Figure 4).

## Discussion

All the AMs whose mRNAs were detectable in various tissues of medaka (Figure 1) are thought to act as paracrine/autocrine factors [10], although a certain amount is secreted into the circulation in human (6.6 pM AM1 in plasma [25]). Tissues showing marked expression of each AM isoform are different among teleost species (Figure 1): AM3 is prominently expressed in the liver and kidney of medaka and eel [6], but mainly in the brain-eye of pufferfish [2]. Conspicuous expression of AM5 was also observed in the liver and kidney of medaka, in contrast to eel and pufferfish in which AM5 is primarily expressed in the spleen and the red body [2,6]. These variations in different organs may reflect the functional versatility of multiple AMs. Although the highest levels of AM3 and AM5 expression in the liver may play a role in growth/nutritional metabolism, these hepatic hormones may play a minor role during adaptation to different salinities, since there were no significant changes in their levels after transfer to freshwater or seawater. However, further studies of AM3/AM5 may reveal the hepatic contribution to osmoregulation. In contrast, the high expression levels of AM1 and AM4 in the kidney as well as AM2 in the gill and kidney suggest that these three AMs could mainly play osmoregulatory roles in these tissues. In tissues where the expression levels were low (e.g., the spleen), however, the cellular localization of AMs suggest they may play significant roles.

Teleost AM1 and AM4 genes are paralogs generated from the same ancestor by the teleost-specific (3R) whole genome duplication, even though the sequence identity of the mature AM1 and AM4 peptides is relatively low (40%) [3,26]. In brain-eye, both increased expression of AM1 in freshwater and decreased level of AM4 in 33 ppt seawater (Figure 2) may suggest a function in inhibiting water intake in hypotonic environment, since previous study in the eel has revealed that water drinking was regulated by systemic administration of AMs although the roles of endogenous AMs expressed in the eel brain were unclear [6,13]. In osmoregulatory organs, AM4 rather than AM1 appeared to be responsible for adaptation to different salinities. Branchial AM4 had the highest expression in isotonic saline (11 ppt), which suggests that AM4 is the most important type among AM family in this tissue. This AM4 appears to be important for Na^+^/K^+^-ATPase activity in the gill of medaka, which was also highest in isotonic saline one week after the transfer and might be involved in both salt absorption and secretion at isotonic salinity rather than having no function [21]. Renal AM4 exhibited increased mRNA levels in freshwater and may also increase Na^+^/K^+^-ATPase activity and sodium absorption in the kidney, because the kidney of fish in freshwater absorbs electrolytes via this enzyme [27].

Similarly to AM1 and AM4, teleost AM2 and AM3 genes were generated by 3R genome duplication with 59% sequence identity of mature peptides in medaka, and are orthologs of mammalian AM2 [3]. We found that expression of AM2 was regulated in the osmoregulatory organs following transfer to different salinities in contrast to AM3 whose expression decreased only in the brain-eye after the transfer to 33 ppt seawater (Figure 3). The upregulation of AM2 in the gill at 33 ppt seawater may suggest a role of sodium excretion into hypertonic environment. In the kidney, unlike AM4, AM2 expressions were inhibited in both freshwater and 33 ppt seawater, suggesting that renal AM2 may be important in the isotonic environment and regulate urine volume and/or urinary sodium excretion. In the eel, AM2 decreases both urine volume and sodium excretion by systemic administration [13]. On the other hand, the AM3 in the brain-eye may inhibit hypoosmoregulation, via suppression of water intake as described for the central AM1/AM4, as well as via regulation of pituitary hormones. In rat, AM2 induces secretion of prolactin [28] and inhibits secretion of growth hormone [29], which are freshwater and seawater-adapting hormones, respectively, in fish [22].

Since the mRNA level of AM5 in brain-eye was low in 33 ppt seawater (Figure 4), AM5 may also attenuate hyperosmotic adaptation by inhibiting water intake like the other AMs. A similar expression pattern of renal AM5 also supports roles for AM5 in inhibition of hypo-osmoregulation as those for AM4. Cardiovascular actions of exogenous AM5 in rat have been described [12], but much of the AM5 function remains unknown, including that in mammalian body fluid regulation.

The regulations of most AM-family gene expressions in the brain-eye and kidney, as well as in the spleen, suggest their involvement in promotion of hyper-osmoregulation and/or inhibition of hypo-osmoregulation in medaka. In our previous study in eel, however, AM2 and AM5 induced water intake by administration into the dorsal aorta [13] though the endogenous source of circulating AMs was unclear. Studies will need to be conducted to determine whether the roles of AM family hormones are conserved among species or AMs have evolved to have diverse functions in different species. To acquire a clearer picture of the physiological roles of the AM family, future studies should focus on identification of their receptors, especially the ligand specificities and the distributions with those of ligands. At present, in teleosts, Nag et al. have identified three orthologs of the mammalian AM (AM1) receptor components, calcitonin receptor-like receptor, as well as five types of receptor activity-modifying proteins in *Takifugu obscurus* [30], yet the binding specificities remain unclear.

## Conclusions

In the present study, the mRNA levels of five AM genes after transfer of medaka to hypo/hypertonic environments suggest that each AM member may play a distinct role in particular organs during adaptation. AM2 and AM4 mRNAs exhibited remarkable changes in the gill and kidney during salinity challenge, suggesting that they play important roles in osmoregulation.

### Availability of supporting data

The data sets supporting the results of this article are included within the article.

## Abbreviations

AM: Adrenomedullin
CGRP: Calcitonin gene-related peptide
